# Evaluation of Cross-Calibrated ^68^Ge/^68^Ga Phantoms for Assessing PET/CT Measurement Bias in Oncology Imaging for Single- and Multicenter Trials

**DOI:** 10.18383/j.tom.2016.00205

**Published:** 2016-12

**Authors:** Darrin W. Byrd, Robert K. Doot, Keith C. Allberg, Lawrence R. MacDonald, Wendy A. McDougald, Brian F. Elston, Hannah M. Linden, Paul E. Kinahan

**Affiliations:** 1 Department of Radiology, University of Washington, Seattle, Washington;; 2 Department of Radiology, University of Pennsylvania, Philadelphia, Pennsylvania;; 3 RadQual, LLC., Weare, New Hampshire; and; 4 Division of Medical Oncology, University of Washington, Seattle, Washington

**Keywords:** PET calibration, quantitative imaging, phantoms

## Abstract

Quantitative PET imaging is an important tool for clinical trials evaluating the response of cancers to investigational therapies. The standardized uptake value, used as a quantitative imaging biomarker, is dependent on multiple parameters that may contribute bias and variability. The use of long-lived, sealed PET calibration phantoms offers the advantages of known radioactivity activity concentration and simpler use than aqueous phantoms. We evaluated scanner and dose calibrator sources from two batches of commercially available kits, together at a single site and distributed across a local multicenter PET imaging network. We found that radioactivity concentration was uniform within the phantoms. Within the regions of interest drawn in the phantom images, coefficients of variation of voxel values were less than 2%. Across phantoms, coefficients of variation for mean signal were close to 1%. Biases of the standardized uptake value estimated with the kits varied by site and were seen to change in time by approximately ±5%. We conclude that these biases cannot be assumed constant over time. The kits provide a robust method to monitor PET scanner and dose calibrator biases, and resulting biases in standardized uptake values.

## Introduction

Cancer is formidable in its resistance to curative efforts. Therapies that prolong survival by a few weeks and cause tumor shrinkage in only 10%–15% of patients are widely prescribed (1). Although there is clear room for improvement in therapy efficacy, the cost of discovering new treatments is high, as many prospective treatments fail in the expensive later phases of clinical trials (2). Positron emission tomography (PET) combined with X-ray computed tomography (CT) (PET/CT) imaging has become a standard component of cancer care management (3–6). This is primarily because of the increased uptake of the PET radiotracer 2-deoxy-2-[^18^F] fluoro-D-glucose (FDG) by many cancer types (3, 4). PET/CT imaging, through the evaluation of the response of cancers to investigational therapies, is being used to accelerate early-phase clinical trials and improve efficiency (7–11).

PET scanners measure radioactivity concentration (kBq/mL). In clinical PET imaging, however, tumor uptake is most often quantified by the standardized uptake value (SUV) within a specified region of interest (ROI). The SUV metric is a scaled measure of tracer concentration that accounts, at least to first order, for variations in the injected amount of radiotracer and patient size (12). The basic expression for calculating SUV (g/mL) is as follows:

(1) $$\text{SUV}=\frac{A}{I/W},$$

where *A* is the decay-corrected radioactivity activity concentration (kBq/mL) measured by the PET scanner within an ROI, *I* is the decay-corrected amount of injected radiotracer (MBq), and *W* is the patient weight (kg), which is used as a surrogate for a distribution volume of the tracer. For reference, if all injected radiotracer were uniformly distributed throughout the body, the SUV everywhere would be 1 g/mL regardless of the radiotracer amount injected and the patient size.

The calculated SUVs are dependent on several parameters that may contribute bias and variability. These sources of error are often poorly controlled in clinical practice (13, 14). One reason for this is that biases in image values do not affect most clinical assessments for either detection or staging (15). However, for tracking disease progression or response to therapy, the quantitative accuracy of PET SUVs is important (6). This issue has been addressed by several recent national and international initiatives, including the Quantitative Imaging Biomarker Alliance (QIBA) FDG-PET/CT Profile (16), the Uniform Protocol for Imaging in Clinical Trials (UPICT) (17), and the European Association of Nuclear Medicine (EANM) procedure guidelines (18).

It is often assumed that in measuring relative changes in SUVs obtained during a baseline study, the impact of most factors affecting calibration in response studies is minimized (ie, canceled out) (19). However, a recent study using a new long-lived calibration source found that biases in SUV measurements varied over time and between scanner sites (20, 21). These results showed that independent dose calibrator and scanner calibration monitoring could be helpful for quality assurance and control. This was also proposed in the FDG-PET/CT profile (16). We will refer to this method as “independent cross-calibration” to distinguish it from methods in which scanner calibration is checked with activity values measured with the on-site dose calibrator.

The present study evaluates a recently designed commercial kit for independent PET/CT cross calibration (X-Cal kit) called the PET ^18^F X-Cal System (RadQual, LLC, Weare, New Hampshire), designed to allow the monitoring of biases in SUV values by enabling the monitoring of biases in equation 1(20). The kits, which contain sealed, long-lived germanium-68/gallium-68 (^68^Ge/^68^Ga) in an epoxy matrix, were subjected to tests to evaluate the repeatability and reproducibility of their measurements, including tests on multiple makes of scanners and dose calibrators across a network of local PET imaging centers.

## Methodology

### Cross-Calibration Kits

Each X-Cal kit contains 3 sealed ^68^Ge/^68^Ga sources for use in a PET/CT scanner, dose calibrator, and well counter (20). Each source's activity is known to within ±2.5% with a 95% confidence level (22, 23). The dose calibrator reference sources are directly traceable to National Institute of Standards and Technology (NIST) standards. The scanner and well counter sources are implicitly NIST-traceable, that is, they are made following the same procedures but are not certified by NIST. Results from the well counter sources are separately reported (24). The present study uses only the scanner source and dose calibrator source from each kit. Two batches from separate manufacturing runs were tested. Batch 1 was manufactured on June 24, 2011, and contained 4 kits, and batch 2 was manufactured on March 3, 2013, and contained 10 kits.

**Figure 1.**
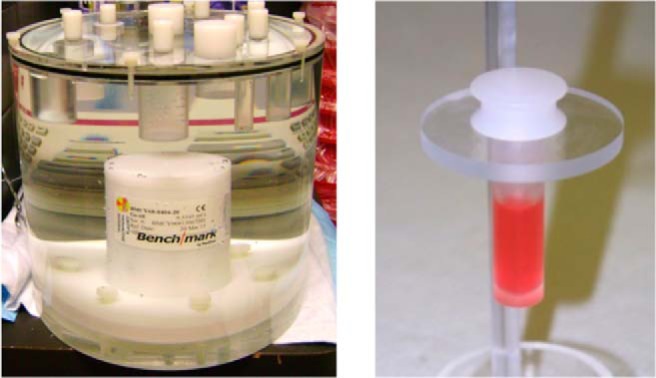
The X-Cal phantom mounted in a 20-cm-diameter water-filled phantom. For all phantom measurements, a PET flood phantom from the American College of Radiology was used after removing the bottom “cold rod” component. No radioactivity is present in the aqueous background except where noted (Left). The National Institute of Standards and Technology (NIST)-traceable dose calibrator source loaded into the clear plastic device used for positioning positron emission tomography (PET) doses inside dose calibrators (Right).

Each scanner source, or X-Cal phantom, has a cylindrical active region with a diameter and height of 45 mm sealed in a non-radioactive acrylic body (Figure 1, Left). The nominal radioactivity and concentration in the phantom are 20 MBq and 250 kBq/mL, respectively. For every measurement in this study, the X-Cal phantom was mounted to the bottom of a 20-cm water-filled flood phantom from the American College of Radiology (ACR), with a special bracket supplied with the kits as shown in Figure 1. The dose calibrator source (Figure 1, Right) contains an NIST-traceable quantity of radiotracer in epoxy (nominal activity 0.90 MBq) in a sealed plastic shell. The dose calibrator source is cylindrical, and it is approximately the same dimensions as a syringe containing a clinical dose of FDG. It is 12 mm in diameter.

### Phantom Density Consistency

A nonradioactive epoxy volume used in the sources was mixed according to the manufacturer's directions, and its density was recorded 7 and 147 days after mixing. Density was calculated by weighing the epoxy in air and under water. In addition, high-resolution CT scans of a single phantom were acquired 2.8 years apart (at 37 and 1061 days after the manufacture date), and a 3-dimensional (3D) segmentation algorithm in the OsiriX DICOM viewer (25) was used to determine the epoxy volume in both scans.

### Phantom Evaluations

All 14 X-Cal phantoms (4 in batch 1 and 10 in batch 2) were scanned on a PET/CT scanner (General Electric Discovery STE, General Electric Healthcare, Waukesha, Wisconsin) at the University of Washington Medical Center. These initial characterization scans were performed on July 31, 2011, for batch 1 phantoms (age 37 days) and between April 1, 2013, and July 9, 2013, for batch 2 (age 12–111 days). The phantom assembly was centered in the field of view and its axis was aligned with the axis of the scanner. A CT scan of the phantom was performed before the PET acquisition. CT images were used for attenuation correction and to check for PET/CT alignment.

Data were acquired in 3D acquisition mode with a single-bed position and a duration of 5 minutes. Scanner corrections were calibrated for ^68^Ge/^68^Ga nuclides. Images were reconstructed by the ordered-subsets expectation maximization algorithm with 4 iterations and 28 subsets. Smoothing after imaging was done by convolution with a 3D Gaussian kernel having a width of 8 mm in the 2 transaxial dimensions and 4.6 mm in the axial dimension. Voxel size was 5.5 × 5.5 × 3.3 mm^3^ for all reconstructions in batch 1 and 2.7 × 2.7 × 3.3 mm^3^ for all reconstructions in batch 2.

#### Short- and Long-Term Variability.

Repeat measurements were performed on a single phantom without repositioning. These data were successively acquired as a dynamic scan having 20 frames, each 5 minutes in length. A single phantom from batch 1 was measured over the course of 238 days (N = 34 acquisitions, phantom age 320–558 days). Coincidence detection rates, which are checked as part of standard daily quality control procedures, and the scanner calibration constant, called the activity calibration factor (ACF), were recorded for each scan. The ACF is what the scanner uses as a global scale factor and is recalibrated on a quarterly basis (26).

### Phantom Dependency Tests

A single phantom from batch 1 was measured with systematic mispositioning. The phantom was rotated in 5 different positions from 0° to 90° (age 236 days). Separately, it was translated directly upward in the field of view in 5 positions over 40 mm (age 475 days). The X-ray tube voltage used in CT scans was set to 80, 100, 120, and 140 kVp. A single PET acquisition was then reconstructed with these 4 CT images used for attenuation correction (age 181 days). On the same day, the background medium in the 20-cm flood phantom was varied with a single X-Cal phantom attached. The 20-cm phantom was filled with the following 3 different background materials: FDG with activity concentration ∼15% of the phantom's concentration, nonradioactive water, and air.

### Dose Calibrator Standard Consistency Tests

On a single dose calibrator (Capintec Radioisotope Calibrator, Model CRC-127R, Capintec Inc., Florham Park, New Jersey), sources from batch 2 were measured (ages 12–111 days). The assay was performed by placing the source (Figure 1, Right) in the syringe holder of the dose calibrator and assaying it as if it were a patient dose using the calibration setting for FDG. The expected reading is computed using a relative response factor that converts the manufacturer's stated activity of ^68^Ge to equivalent ^18^F activity (22, 23).

### Multisite Testing

X-Cal kits were distributed to 6 sites of a local cancer center network (Seattle Cancer Care Alliance Network), along with protocols for measurements. Seven PET scanners were used, as 1 site upgraded to a newer scanner during the study. University of Washington personnel visited each participating site for a review of equipment and procedures before the first scan. Sites used their clinical whole-body protocol to scan the phantom and default dose calibrator settings to assay the dose calibrator source. A total of 24 calibration pairs of phantom and dose calibrator measurements were recorded over a 2.6-year period (age, 248–1186 days). The data acquisition protocol was as follows:

(1) The dose calibrator source is measured in the dose calibrator.
(2) All clocks used are synchronized with the PET scanner ±1 minute.
(3) The PET scanner phantom is mounted in a standard 20-cm water-filled phantom using an attachment plate supplied with the X-Cal kit as shown in Figure 1. There is no radioactivity in the water.
(4) The radionuclide for the scan is set to ^68^Ge.
(5) The X-Cal phantom is coaxially aligned and centered in the PET scanner. The phantom is then scanned and PET and CT images reconstructed using the site's standard whole-body (nonbrain) oncology PET/CT protocol.
(6) The PET and CT imaging parameters are recorded.

### Image Analysis

All PET image metrics were calculated using the “XCaliper” automated analysis package. XCaliper is a plug-in tool for the OsiriX DICOM viewer (25) (see online Supplemental Appendix for full details on the algorithm and usage). Here, we note that XCaliper is fully automated and uses the OsiriX viewer to provide a visual check that the PET and CT images are aligned and that the ROI is drawn in the correct location. When XCaliper is activated, it finds the center of the active area of the phantom (without user intervention) and computes values from an ROI of only whole voxels that fit inside a 1.5-cm^3^cubic bounding box. The size of the bounding box was chosen to be small enough that it was not sensitive to partial volume effects at the edges of the phantom (see online Supplemental Appendix). Larger and smaller bounding boxes were used for batch 2 images to test for ROI size dependence. The values reported by OsiriX from the ROI are mean and standard deviation of included voxels and maximum and minimum voxels, reported in both activity concentration (kBq/mL) and weight-based SUV (g/mL).

For our analysis of PET phantoms, we report results as the dimensionless recovery coefficient *R* [equation 2], defined as the ratio of the measured values to the known value and is calculated as follows:

(2) $$R=\frac{A_{M}}{A_{K}}$$

where *A_M_* is the measured radioactivity concentration (kBq/mL), averaged over voxels in the ROI, and *A_K_* is the known NIST implicitly traceable concentration. A subtle point is that we do not call the latter the “true” value, as the NIST-traceable standards are stated with a known measurement uncertainty of ±2.5% with a 95% confidence level (22, 23). Where needed, a similar ratio measurement was used for the dose calibrators. In this case, however, the dose calibrators measured total activity, as opposed to concentration. We use the coefficient of variation (COV), which is the standard deviation divided by the mean, to characterize the variability of *R* across independent measurements. In addition, we calculate *b*, the bias in SUV values described by Doot et al. (21), as $b=(R_{P}/R_{D})-1$. Here, *R*_*P*_ and *R*_*D*_ are the recovery coefficients for the PET phantom and the dose calibrator sources. We also report *u*, the coefficient of variation of voxel values within the ROI.

## Results

### Phantom Density Consistency

Epoxy densities calculated from weight measurements in air and water are shown in Table 1. A small but statistically significant difference was observed in the 2 measurements (*P* = .04 with paired, 2-tailed *t* test) equal to a 0.09% increase in the epoxy density.

**Table 1.** Measured Epoxy Density

Cure Time (days)	Density (g/mL)
7	1.1404 ± 0.0025
147	1.1414 ± 0.0024

**Figure 2.**
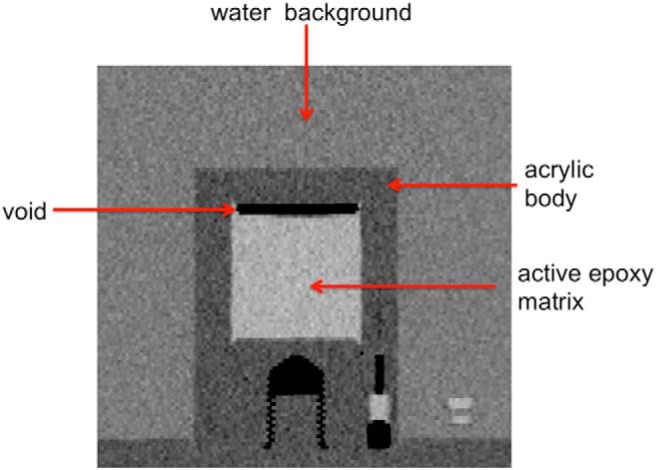
Coronal section from a computed tomography (CT) image of the X-Cal phantom in a water-filled flood phantom.

High-resolution CT scans of a phantom from batch 1 showed that a void was present within the phantom's interior, visible in Figure 2. The epoxy volume, computed by segmentation, was 66.78 and 67.64 mL at 37 and 1061 days, respectively, after manufacture. These measurements differ by 1.3%

### Phantom Evaluations

**Figure 3.**
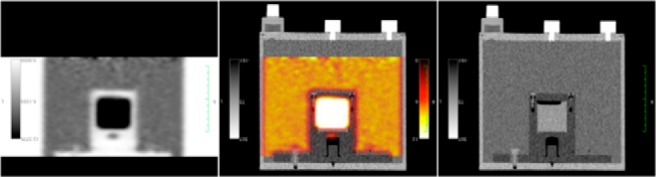
PET, PET + CT fused, and CT image of the X-Cal PET phantom inside a 20-cm-diameter water-filled phantom. In this case, a small amount of FDG was added to the background water.

**Figure 4.**
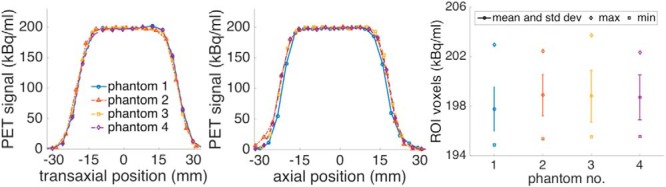
PET data from 4 phantoms of Batch 1. Transaxial profiles averaged over rows included in the XCaliper ROI (Left). Axial profiles with values averaged over the 4 voxels corresponding to the transaxial location of the ROI (Center). Distribution of all voxel values in the ROI (Right).

An illustrative image of the PET and CT and fused images is shown in Figure 3. PET image profiles and voxel value distributions from the 4 phantoms of batch 1 are shown in Figure 4. The summary statistics for both batches of X-Cal PET phantoms are listed in Table 2.

**Table 2.** Measured *R*s for the X-Cal PET Phantoms

Batch	Date	N	*R* mean	*R* SD	*u* (%)
1	2011	4	0.95	0.006	0.92
2	2013	10	0.92	0.011	1.28

The last column is the average COV of the voxel values in the ROIs*, u,* and is a metric of spatial uniformity.

#### Short-Term Variability.

*R* has a COV of 0.36% over 20 five-minute successive acquisitions with no repositioning.

#### Long-Term Variability.

**Figure 5.**
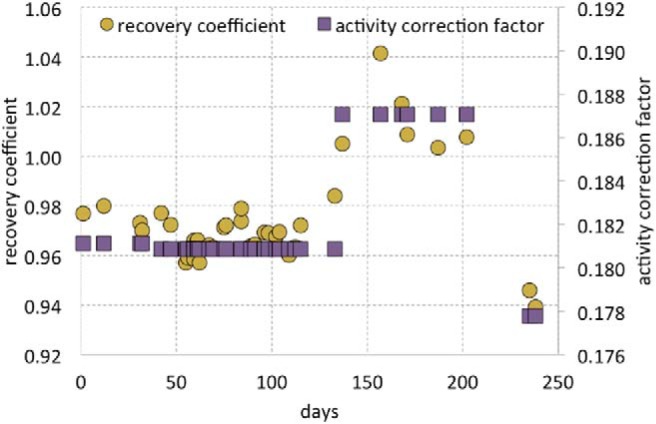
Time series of X-Cal phantom recovery coefficients (*R*) and scanner activity calibration factors (ACFs) over 8 months. The 3 shifts in ACF values are because of quarterly scanner calibrations following the manufacturer's standard procedures.

The time series of X-Cal *R*s and ACFs over 8 months is shown in Figure 5. Changes in the ACF from quarterly calibrations on the PET scanner are apparent. Over 8 months on a single scanner, *R*s for a single source had a range of 0.939 to 1.042 and a standard deviation of 0.021 (COV of 2.1%). Taking a subset of the data from a single calibration period (experiment days 42 through 133) reduces this range from 0.957 to 0.984 with a standard deviation of 0.007 (COV of 0.7%).

### Phantom Dependency Tests

**Figure 6.**
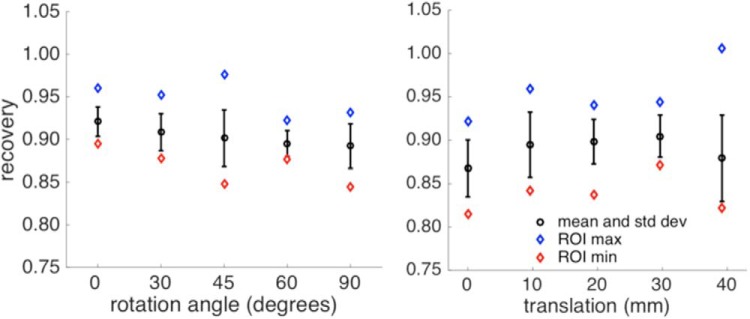
XCaliper ROI voxel distribution for the rotated (Left) and translated (Right) scans of the X-Cal phantom. Here, the y-axis represents measured/known for individual voxel values.

Figure 6 shows the voxel distributions from the rotation and translation scans. *R* ranged from 0.87 to 0.92. *R* distributions were 0.889 ± 0.015 for translations and 0.903 ± 0.012 for rotations (COVs of 1.7% and 1.3%, respectively).

**Figure 7.**
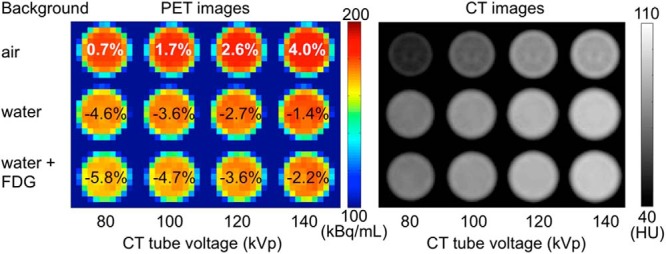
Changes in signal from PET images as background media and CT tube voltage are varied. The CT images on the right were used for attenuation-correction in the generation of the corresponding PET images on the left. Percentage values over the PET images are the biases measured for the central XCaliper region of interest (ROI) versus known activity concentration.

Changing CT tube voltage and background media caused changes in the bias of PET images, as seen in Figure 7. Changing tube voltage from 80 to 140 kVp caused *R*s to go from 0.968 to 1.000 (averaged over backgrounds), whereas changing background medium from air to water to radioactive water changed bias from 1.023 to 0.969 to 0.959, respectively (averaged over CT tube voltages). The overall COV was 3.2% across all variations.

### ROI Size Tests

Changing the size of the bounding box in XCaliper from 10 to 15 to 20 mm in the images of batch 2 sources led to ROIs with 27, 100, and 294 voxels, respectively. A paired, 2-tailed *t* test showed no significant differences between the small and medium ROI mean values (*P* = .14) or between the medium and large values (*P* = .26).

### Dose Calibrator Standard Consistency Tests

Dose calibrator standards from batch 2 (N = 10) measured 1.005 ± 0.0039 (COV = 0.39%) in units of total assayed activity over known activity.

### Multisite Testing

**Figure 8.**
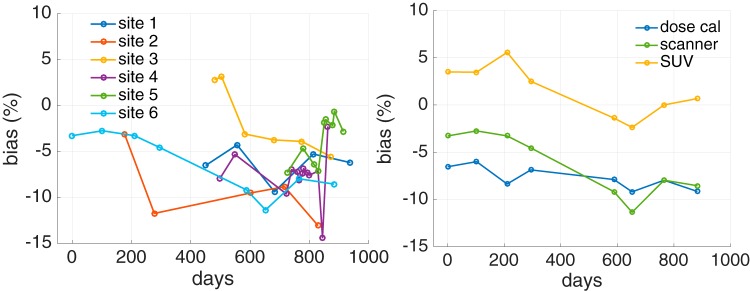
PET scanner bias versus time from 6 network hospitals. Scans were collected from the 6 sites over 2.6 years. Overall, the ensemble *R*s had a mean value of 0.944 ± 0.038 (Left). Scanner, dose calibrator, and resultant SUV bias versus time for a single site showing variable SUV bias in time (Right).

Across a local network of imaging centers, per site average *R*s ranged from 0.907 to 0.983, with per site standard deviations between 0.019 and 0.034. The measurements overall had a mean of 0.944 ± 0.038 (COV = 4.0%) (Figure 8, Left). Dose calibrator recovery coefficients were 0.964 ± 0.033 (COV = 3.4%).

For a single site, Figure 8 (Right) shows the estimated SUV bias, which varies in time. A comparison of the per test PET scanner and dose calibrator biases did not show any correlations in the biases (data not shown).

## Discussion

Long-lived, sealed PET calibration phantoms offer the advantages of being simpler to use than aqueous phantoms and the potential of known radioactivity concentrations (27). The X-Cal independent cross-calibration kits were developed based on the traceable dose calibrator standards from RadQual, LLC., and the NIST (22, 23). In a previous study, using a larger prototype phantom with a 6 cm diameter, we found that bias in SUV measurements varied over time and between scanner sites (21). Those results demonstrated that the use of dose calibrator and scanner cross-calibration kits could be helpful for quality assurance and control. The results of this study confirm those results, as described below, and provide a systematic approach.

Physical measurements and CT imaging of the epoxy showed the material to be reasonably stable in time in its density (Table 1) and volume. The manufacturer of the epoxy states that it should be fully cured after 2 days at room temperature. The small detected changes in volume should not unduly affect the phantom performance, as they are smaller in magnitude than the scanner calibration changes they are intended to detect, such as those in Figure 5. During phantom manufacture, it is the activity per gram that is known with precision. Consequently, the physical density of the cured epoxy enters linearly into the determination of activity per volume. The stability of the epoxy density ensures that the activity density is constant in time.

The 4.5-cm-diameter active region is large enough to draw a many-voxel ROI that does not suffer from resolution losses and high enough in radioactivity concentration to provide a signal that is not adversely affected by quantum noise (Figure 4). Our testing showed that ROI mean values were stable for ROIs both larger and smaller than our recommended ROI size. Activity concentration was uniform within the phantoms (Figure 4). Within the ROIs drawn by XCaliper, COVs of voxel values were <2%, and profiles through the phantoms showed good agreement. Phantom uniformity is an important characteristic, as variations in activity concentration may lead to increased variability of ROI means if, for instance, phantom repositioning leads to different voxel and ROI positions within the active region. Variability between phantoms was low, with COVs close to 1% for each batch's *R*s (Table 2). A slight difference was observed between batches, with batch 1 having a mean of 0.92 and 0.95 for batch 2. This small disparity could be due to differences in phantom manufacturing or changes in the calibration of the PET scanner used, as the measurements were made 2 years apart. The short-term variability was smaller than any other variability tested, with repeat scans having a COV of 0.36%. This contribution of quantum noise to *R* variability should remain small under clinical conditions, and likely can be reduced by scanning longer to collect more counts as shown by Doot et al. (28). Measurements of a single phantom and scanner parameters over 8 months showed that *R* values of the phantom were correlated with the ACF of the scanner (Figure 5). This is expected, as the scanner uses the ACF as a global scale factor (26). Although the overall variability was high for the full-time series (average COV of 2.1%), the variability for a 3-month period where the ACF was not changed was 3 times lower (average COV of 0.7%). Our interpretation of these results is that the inter- and intra-phantom variability for the X-Cal PET phantom was below what could be measured with the PET scanner itself.

The effect of translations and rotations on measurements of the X-Cal phantom was more a test on the robustness of the XCaliper analysis program. Given that these mispositionings likely spanned a greater range than would be reasonably expected in typical phantom use and that *R* ranged from 0.87 to 0.92, we conclude that the phantom performance, in conjunction with the XCaliper plugin, is unlikely to be affected by operator variations in positioning.

The dose calibrator sources had very low intra- and inter-batch variability and bias. These results are in agreement with those of previous studies (22). Because these sources were made from the same epoxy batch as the X-Cal phantom batch, their activity was constrained by the concentration selected for the phantom. This meant that the radioactivity concentration in the sources was lower than typical clinical oncology values. Our work has assumed, but not verified, that the dose calibrator's response is linear (ie, accurate) over a range that spans the activities of our sources and clinical values. The constancy of the dose calibrator bias as the sources decayed is consistent with this assumption. In contrast, it has been shown that the scanner sources are well within linear response ranges for modern PET/CT scanners (29–31).

The change in PET values seen in Figure 7 shows that bias is dependent on both the background in the 20-cm-diameter phantom and the CT tube voltage. The former effect is likely because of variations in the accuracy of the scatter estimation, whereas the latter effect is known to be because of variations in accuracy of the CT-based attenuation correction (32). We chose to have a water background to reduce potential challenges for a PET scanner's scatter estimation algorithm. A full consideration of these effects is beyond the scope of the present work. However, these results illustrate that although the radioactivity concentration in the phantom is known with a high degree of precision, the measurements will have a bias that can vary with the radiotracer environment and CT imaging methods.

Testing the cross-calibration kits in our local network sites showed that PET scanner biases were surprisingly variable across sites and in time (Figure 8, Left). We note that in one instance, use of the X-Cal phantom led to the discovery of a misalignment of 7 mm between the PET and CT images. Extensive discussions were held with the hospital and the scanner manufacturer to determine the root cause. After subsequent testing and exchanges of image data with the manufacturer's engineering team, the cause was determined to be mechanical distortions of the floor on which the scanner had been mounted. The issue was resolved and the site resumed scanning the phantom with correct alignment.

Tests of the variability of the dose calibrator measurements from multiple-dose calibrators have been presented elsewhere (33), and it was found that there was both bias and variability in the reported values for 39 dose calibrators at 3 institutions. In our data, SUV biases were also seen to change in time by greater than ±5% as calculated from dose calibrator and scanner biases (Figure 8, Right). This suggests that the process of using the same dose calibrator for scanner calibration and for patient dose assays does not cancel out biases in SUV estimation, which has been suggested (19). This increased variability is consistent with the variability in clinical patient SUV values reported by Kumar et al. (34). It appears that scanner biases are more variable than dose calibrator biases. The intuitive argument that their biases should cancel from SUVs does not take this greater variability into account and, thus, risks oversimplifying SUV behavior.

Recently, it has been shown that variability across a network of hospitals can vary based on whether long-lived ^68^Ge/^68^Ga or short-lived^18^F sources are used (35). We note that there is a subtlety in quality control of SUVs, in that short-lived phantoms can detect more sources of variability but cannot separate them. Long-lived phantoms can separate bias instability in the scanner and dose calibrator and follow extremely stable decay curves, but may miss effects such as clock synchronization or incomplete dose injection. Short-lived phantoms are commonly used to assess scanner bias for clinic accreditation (35–37), and in 1 such study, 12% of scanners tested (N = 101) were found to have biases >10% (36). This shows that quality control for PET is essential, and we believe that long-lived sources have a key role to play in characterizing SUV variability in time.

In all, we saw the largest COVs for measurements that spanned sites (4.0%). The single-scanner COV was about half of that with recalibrations (2.1%) and smaller still for a single calibration period (0.7%). Improper positioning of the phantom led to COVs between 1% and 2%, whereas quantum noise was responsible for the smallest COV of 0.36%.

The *R* values measured at our own site and at network hospitals were less than the expected value of 1 in a large majority of cases. Previous work has shown that absolute signal recovery is confounded by mismatches in physics modeling between patients and the phantom, in particular for attenuation correction for photons absorbed in the epoxy (32). We expect that carefully filled aqueous phantoms scanned at these sites would recover known signals with better accuracy than those seen in the phantoms. However, constancy of bias may still be monitored with these phantoms, which, for test–retest studies, may be of greater importance than absolute calibration accuracy. The high degree of uniformity (Figure 4 and Table 2) and the high correlation with the activity correction factor (Figure 5) make the X-Cal phantom a viable tool for the monitoring the constancy of PET scanner calibration biases.

The X-Cal kits, combined with the Xcaliper plugin for OsiriX, provide a robust method to monitor scanner, dose calibrator, and SUV biases. Although absolute biases in PET/CT patient scans will be subject to different biasing factors because of physics, these physical biases are not likely to change for a given patient or scanner. The X-Cal kits are therefore well suited to monitor changes in instrument calibration that will lead to changes in SUV bias.

### Supplemental Materials

Supplemental Appendix:
