# Supplementary material for: Evaluation of Cross-Calibrated 68Ge/68Ga Phantoms for Assessing PET/CT Measurement Bias in Oncology Imaging for Single- and Multicenter Trials
Source: Tomography. 2016 Dec;2(4):353–60. doi: 10.18383/j.tom.2016.00205 (PMC5214172; doi:10.18383/j.tom.2016.00205)
Supplement: Supplemental Appendix: [file tom-00205-16-s001.pdf]

Appendix

University of Washington XCaliper

XCaliper is an Osirix plug-in for automated measurement of radioactivity concentrations from the X-Cal PET phantom. Available at http://depts.washington.edu/xcaliper.

The purpose of the XCaliper plug-in is to place a 3-D ROI in a PET image of the RadQual X-Cal phantom as shown in Figure A1. The ROI includes only whole voxels and is centered on the small (4.5 cm diameter) active cylinder containing $^{68}Ge/^{68}Ga$ in epoxy.

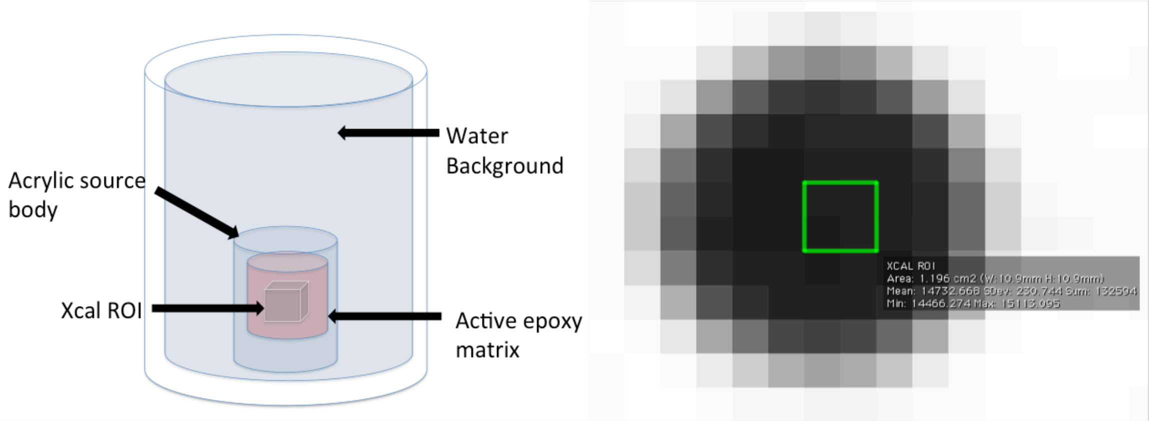

Figure A1. Left: Diagram of RadQual's PET ^{18}F X-Cal scanner source, showing the ROI in the center of the active region. Right: transaxial section of the PET image of X-Cal phantom. A 2D cross section of the automatically selected voxels are indicated by the locus of the green box. This transaxial span is propagated through several slices, and XCaliper returns metrics from the entire volume of interest.

The localization algorithm uses an automated centroid-finding algorithm for repeatable and user-independent placement. After localization and an internal consistency check on the segmented phantom volume, properties of the PET image inside the ROI are calculated. These include, but are not limited to, mean activity (kBq/ml), maximum and minimum activity, standard deviation, and volume. ROI metrics for the entire volume are displayed in an OsiriX window and written to an XML file. For a representative included slice, OsiriX displays a planar ROI to indicate the voxels selected by the algorithm, as shown in Figure A1 (right).

The algorithm operates by first taking the active part of the phantom to be the set of voxels whose values exceed the user-defined threshold. This segmented phantom image is defined as $p_{ijk} = 1 \forall \left\{ i, j, k \mid a_{ijk} > t \right\}$, and zero otherwise,

where $t = T \cdot \max\{a_{ijk}\}$, T is a threshold between 0 and 1, and a_{ijk} is the array of image voxels. The ratio of the computed volume to expected volume is then used as a check of whether the segmentation performed as intended. The centroid of the phantom is then computed, and the number of whole voxels to be included in each dimension is calculated based on the user-specified maximum dimensions and voxel size.
